# Adolescent Understanding and Acceptance of the HPV Vaccination in an Underserved Population in New York City

**DOI:** 10.1155/2012/904034

**Published:** 2011-12-11

**Authors:** Jill Blumenthal, Melissa K. Frey, Michael J. Worley, Nana E. Tchabo, Karen Soren, Brian M. Slomovitz

**Affiliations:** ^1^Department of Internal Medicine, New York Presbyterian Hospital-Weill Cornell Medical College, New York, NY 10021, USA; ^2^Department of Obstetrics and Gynecology, New York Presbyterian Hospital-Weill Cornell Medical College, 525 East 68th Street, New York, NY 10021, USA; ^3^Department Obstetrics and Gynecology, Brigham and Women's Hospital, Boston, MA 02115, USA; ^4^Department of Obstetrics and Gynecology, Morristown Memorial Hospital, Morristown, NJ 07960, USA; ^5^Department of Pediatrics, Columbia University Medical Center and New York Presbyterian Morgan Stanley Children's Hospital, New York, NY 10032, USA

## Abstract

*Background*. HPV vaccination may prevent thousands of cases of cervical cancer. We aimed to evaluate the understanding and acceptance of the HPV vaccine among adolescents. *Methods*. A questionnaire was distributed to adolescents at health clinics affiliated with a large urban hospital system to determine knowledge pertaining to sexually transmitted diseases and acceptance of the HPV vaccine. *Results*. 223 adolescents completed the survey. 28% were male, and 70% were female. The mean age for respondents was 16 years old. Adolescents who had received the HPV vaccine were more likely to be female and to have heard of cervical cancer and Pap testing. Of the 143 adolescents who had not yet been vaccinated, only 4% believed that they were at risk of HPV infection and 52% were willing to be vaccinated. *Conclusions*. Surveyed adolescents demonstrated a marginal willingness to receive the HPV vaccine and a lack of awareness of personal risk for acquiring HPV.

## 1. Introduction

Approximately 20 million people in the United States are infected with genital human papillomavirus (HPV) [1]. An estimated 5.5 million people will acquire a new genital HPV infection each year, and the incidence of infection is highest among sexually active adolescent girls and young women between the age of 18 and 28 years old. It has been reported that 37% of males and 28% of females in the ninth grade have had sexual intercourse and 7% of students had sexual intercourse before the age of 13 [2]. Therefore, HPV vaccination ideally would be directed toward preadolescents and young adolescents in an effort to provide the greatest public health benefit offered by a prophylactic HPV vaccine.

It is predicted that HPV vaccination will prevent thousands of cases of cervical cancer worldwide. In addition, the emotional stress and economic burden associated with abnormal Papanicolaou test results and the treatment of preinvasive cervical lesions will be greatly reduced. The quadrivalent HPV vaccine (Gardasil, Merck & Co.) was found to have 97% efficacy at preventing HPV 16 and/or 18 related cervical intraepithelial neoplasia 2 or 3, adenocarcinoma in situ, and cervical cancer [3]. The quadrivalent HPV is approved for use in males and females between 9 and 26 years of age. On October 16, 2009, the FDA approved a recombinant HPV bivalent vaccine (Cervarix, Glaxo Smith Kline Biologicals) to prevent cervical cancer and precancerous lesions caused by HPV types 16 and 18. This HPV vaccine is approved for use in females between 10 and 25 years of age and is the second HPV vaccine licensed for use in females in the United States. 

 Until recently, studies of women and young adults have shown poor levels of knowledge about HPV, Papanicolaou smear testing, and cervical cancer [4, 5]. Current research indicates that understanding has improved. However, knowledge of some relevant issues is higher than others. A review by Brewer and Fazekas reported that only 21% of respondents knew that HPV is common, 59% knew the purpose of a Papanicolaou smear, and 68% knew that HPV is a sexually transmitted infection [6]. In addition, awareness of HPV among a racially diverse sample of young adults, aged 18–26 years old, was found to be relatively high, with more than 75% of study participants indicating that they had heard of HPV from various sources [7]. However, another recent study examining the acceptability of the HPV vaccination among Latina immigrants and African American women found that 61% of Latinas and 78% of African Americans had never heard about HPV [8]. 

 Continuing, several studies indicate that most parents, especially with proper knowledge of HPV and the vaccine, are willing to accept the HPV vaccine for their children [9–12]. Healthcare providers are an important source of information for parents and children, and studies show that they generally have a positive attitude towards recommending HPV vaccination [13, 14]. The vaccination series can be started beginning at 9 years old, and the Advisory Committee on Immunization Practices currently recommends routine vaccination of females aged 11 or 12 years with three doses of HPV vaccine. In addition, the quadrivalent HPV vaccination has been approved in males aged 9 through 26 years to reduce their likelihood of acquiring genital warts. Ideally, vaccination should be administered before potential exposure to HPV through sexual contact [15].

 The purpose of this study was to evaluate the understanding and potential acceptance of the HPV vaccine by adolescents aged 13 to 18 years at adolescent health clinics affiliated with New York Presbyterian Hospital. Although previous research shows that most adolescents, parents, and health care providers are willing to accept universal vaccination for themselves, their attitudes and knowledge regarding the vaccine have yet to be evaluated. Improved knowledge of adolescent understanding and acceptance of the HPV vaccine would help practitioners provide appropriate and useful information to parents, providers, and adolescents when making the decision to vaccinate. 

## 2. Materials and Methods

Approval for this study was received from the institutional review board. We administered a questionnaire to all adolescent patients (defined as between 13 and 18 years of age) awaiting annual well-patient medical examinations in adolescent health clinics affiliated with Weill Cornell Medical College and Columbia University Medical Center. These health clinics provide affordable, comprehensive services to a diverse patient population including underserved populations from each of the five boroughs in the New York City area. The adolescent patient was provided the questionnaire in English and/or Spanish. We could not predict the number of adolescent patients who would be interested in participating in this study, and therefore prior to study initiation we defined the survey collection period as September 1, 2007 through February 1, 2008 with the goal of collecting the maximum number of surveys possible. The research personnel emphasized that participation was voluntary, anonymous, and without incentive for questionnaire completion. 

The questionnaire consisted of two parts; the first part elicited the adolescent's demographic information and knowledge pertaining to sexually transmitted diseases and vaccinations. When the first part was completed, the adolescent was provided with a second part that contained current facts about the HPV vaccine and cervical cancer, followed by questions specifically designed to ascertain adolescent acceptance of the vaccination. Adolescents whose primary language was neither English nor Spanish were excluded from the study since the questionnaire was self-administered and available only in English and Spanish. 

Data were analyzed using commercially available software (SPSS version 16.0; SPSS, Inc., Chicago, IlL). A series of *χ*2 and Fisher's exact tests were used for comparison of proportions between “HPV vaccine status” and 15 other variables of interest (see Table 2) and “Willingness to receive the HPV vaccine” and 15 other variables of interest (see Table 3). A *P* value of  < 0.05 was considered significant for all tests. For the series of 15 analyses, the sample sizes of 223 participants (HPV vaccine status) and 143 participants (willingness to be vaccinated) provided a mean power >99.9% for detecting a difference between both whether the adolescent had been vaccinated and whether the adolescent was willing to receive the HPV vaccine and the respective variable, using 2-tailed test with statistical significance defined as *P* < 0.05.

## 3. Results

Of the 223 adolescents who completed the survey, 28% were male and 70% were female (2% did not respond to the question). The mean age of respondents was 16 years old (range 13–18). The demographics of adolescents including in this study are displayed in Table 1. A majority of adolescents surveyed (168, 75%) stated that they had prior knowledge of cervical cancer. However, a smaller percentage of adolescents (103, 46%) acknowledged prior knowledge of what a “Pap smear” was, and only 57 respondents (26%) were aware that the Pap smear was used to screen for cervical cancer. 5% (11) of respondents reported knowing someone who had/has cervical cancer, and 91% (202) were familiar with the term “sexually transmitted disease” or “STD”. 

Of the 223 adolescent surveyed, 33% stated that they had received the HPV vaccine, 64% had not had the vaccine, and 3% where not sure. Among the 143 adolescents who had not yet been vaccinated, 52% were willing to get the HPV vaccine, 12% were not willing, and 36% were unsure. Of the 143 adolescents who had not yet been vaccinated, only 4% believed that they were at risk for being exposed to HPV.

Of the 73 respondents that stated that they had received the HPV vaccine, the mean age was 16 years old (range 13–18). Female respondents were more likely to have received the HPV vaccine than male respondents (45% versus 3%, *P* < 0.001). HPV vaccination status was not significantly associated with language, race, religion, or having younger or older siblings who had received the HPV vaccine. HPV vaccine status was associated with knowledge of cervical cancer (41% of those who had heard of cervical cancer were vaccinated), knowledge of Pap screening (43% who had heard of Pap screening were vaccinated), and having had other vaccines (73% of those who had received other vaccines were vaccinated) (Table 2).

Among the adolescents who had not yet received the HPV vaccine, 58% of females and 42% of males were willing to be vaccinated; however this difference was not statistically significant. In addition, 53% of Hispanic adolescents and 56% of African American adolescents were willing to accept vaccination. There was no association with willingness to receive the vaccine and gender, race, religion, language, having vaccinated siblings, knowledge of cervical cancer, Pap screening, sexually transmitted disease, or any other studied variable.

## 4. Discussion

The current study reveals only a marginal willingness of teenagers to accept the HPV vaccine. The majority of data to date suggest that young women are interested in vaccines that prevent HPV and other sexually transmitted infections. However, most of these studies have been conducted with women and men of 18 years of age or older. Holcomb et al. found that women are interested in learning about HPV, how the virus is transmitted, and how women can prevent becoming infected [16]. In 2001, a study of women recruited from community and clinical sites displayed that roughly 85% of participants indicated an intention to receive an HPV vaccine for cervical cancer prevention once it became available [17]. A study of young, Australian, men and women aged 18–23 years old showed a high vaccine acceptance despite inadequate knowledge of HPV infection [18]. Another study of male and female college students found an overall acceptance rate of the HPV vaccine of 74%. In this study, although females were more likely to have already been vaccinated, gender did not affect vaccine acceptance among unvaccinated adolescents [19]. In contrast to most of the data available in the literature, this current review sampled a younger population. We found a generalized lack of awareness of HPV among adolescents. Although the majority of adolescents had heard of cervical cancer (75%), only 46% knew what a Pap smear was, and only 26% knew that the Pap smear is a screening test for cervical cancer. Furthermore, only 4% of the adolescents surveyed who had not yet received the HPV vaccine believed that they were at risk for HPV infection. The lack of awareness about cervical cancer screening and underestimated risk of HPV risk likely contributes to the limited willingness of the adolescents surveyed to accept the HPV vaccine for themselves.

Many of the adolescents stated that they would seek advice from a parent or physician when deciding whether or not to accept the HPV vaccine. Interestingly, we found no significant association between having had the HPV vaccine or willingness to receive the vaccine and the level of education of the adolescent's parents. There was also no association between having younger or older siblings who had been vaccinated and having had the HPV vaccine or willingness to receive the vaccine. Many studies have shown that most parents are willing to accept the HPV vaccine for their children. A recent study, postlicensure of the vaccine, evaluated mothers of children in primary and secondary schools in England and found that 75% of mothers would accept the HPV vaccine for their daughter. Acceptance was higher in mothers who had experienced cancer in their families, had older daughters, perceived approval from their husbands/partners, and believed vaccine acceptance would be more normative [20]. Another postlicensure study used a random-digit-dial telephone survey to examine the likelihood of parental acceptance of the HPV vaccination for young adolescent girls in California. Within this sample, 75% of parents were likely to vaccinate a daughter before age 13. Hispanic parents were more likely to accept vaccination than were non-Hispanic parents, and African American and Asian American parents were the least likely to accept vaccination [21]. Overall, parental attitude and acceptance studies suggest a great deal of parental interest in HPV vaccination for their adolescent children. Healthcare providers may be able to encourage participation in HPV vaccine programs by bolstering parents' underlying desire to protect their children, as recommendation by a healthcare provider is a crucial prompt for vaccination.

 Healthcare providers are an important source of information for parents and children, and studies show that they generally have a positive attitude towards recommending an HPV vaccination. One study of 207 fellows of the American College of Obstetricians and Gynecologists found a fairly positive disposition towards recommending the HPV vaccine, with a mean rating for vaccine recommendation of 79 out of 100 [22]. Similarly, 224 nurse practitioners had a favorable attitude about HPV vaccine recommendation, with a mean rating of 72 out of 100 [23]. Our study reveals that knowledge of cervical cancer and Pap screening is associated with having had the HPV vaccine, however interestingly not with willingness to accept the vaccine among those not yet vaccinated.

 Previous studies have shown that knowledge of personal risk is generally limited with regard to sexually transmitted infections. A study of sexually active heterosexual college students found that personal risk of contracting Acquires Immune Deficiency Syndrome was estimated to be significantly lower than risk for each of a set of hypothetical persons who varied in degree of similarity to respondents [24]. In a study of university students in the United Kingdom, screening for chlamydia was limited, and perception of personal risk was poor [25]. In a pilot study among Australian university students, even though the majority of students were comfortable with opportunistic testing for chlamydia by their general practitioner, the likelihood of being tested in the upcoming year for most students was low, as was personal concern about chlamydia infection [26]. With regard to HPV, a recent study of Australian women with diverse sexual orientation, found that the majority of women had risk factors for HPV but few felt personally at risk of acquiring infection [27]. Among the 143 adolescents in our study who had not yet received the HPV vaccine, only 4% believed that they were at risk for being exposed to HPV. The very low personal risk perception for HPV suggests the need for targeted education for this group regarding HPV transmission and prevention.

 Study limitations should be considered when interpreting the present findings. Participants were recruited from a hospital-based adolescent health clinic serving a racially and ethnically diverse, predominantly low-income, population. As a result, the findings may not be applicable to all adolescents in the United States. In order to include 13 and 14 years old in this study at an adolescent health clinic, we were unable to ask certain direct questions, including “Are you sexually active?”. The answer to this question may have provided more information regarding age of sexual activity in the current study population and would allow direct comparison of these results with a personal risk perception question. In addition, because the time frame was not stated explicitly in the question regarding personal risk, it is unclear whether participants were rating their risk of HPV infection at present, in the immediate future, or over their lifetime, which would also affect the accuracy of their ratings. 

## 5. Conclusion

It is clear that education will play an important role in the implementation of an HPV vaccination program. In this study, only 53% of adolescents who had not yet received the HPV vaccine were willing to be vaccinated, and lack of education likely contributes to this hesitancy. Healthcare providers must be proactive in educating and discussing benefits of vaccination. Based on factors affecting vaccine acceptability, HPV vaccine programs in the United States should emphasize high vaccine effectiveness, the high likelihood of HPV infection, physician recommendations, and address barriers to vaccination. It is important to educate patients that the vaccine is most effective prior to sexual activity and HPV exposure. However, the vaccine does not replace routine screening for cervical cancer or the need for education about continued cervical cancer screening. Thus, it is essential for healthcare providers to offer guidance about the ongoing need for screening. Future studies will be necessary to evaluate public health issues that arise once the HPV vaccination programs have been established more extensively.

## Figures and Tables

**Table 1:** Description of adolescent characteristics.

	Variable	*n* (%)
Mean age		15.9 (range 13–18)

Sex		
	Male	62 (27.8)
	Female	155 (69.5)
	Blank	4 (1.8)

Race		
	African American	47 (21.1)
	Native American/Aleutian/Eskimo	1 (0.4)
	Hispanic	145 (65.0)
	Asian	3 (1.3)
	Caucasian	6 (8.5)
	Other	19 (8.5)
	Blank	2 (0.9)

Language		
	English	210 (94.2)
	Spanish	11 (4.9)
	Blank	2 (0.9)

Religion		
	Catholic	132 (59.2)
	Jewish	1 (0.4)
	Buddhist	2 (0.9)
	Protestant	3 (1.3)
	Muslim	8 (3.6)
	Jehovah's Witness	3 (1.3)
	No religion	29 (13.0)
	Other	39 (17.5)
	Blank	6 (2.7)

**Table 2:** HPV vaccine status.

	Have had HPV vaccine		Have not had HPV vaccine		Do not know		*P* value
	#	%	#	%	#	%	
Language							1.000
English	69	32.9%	137	65.2%	4	1.9%	
Spanish	4	36.4%	7	63.6%	0	0.0%	

Age (mean)		16.08		15.85		—	0.311

Gender							<0.001
Male	2	3.1%	60	93.8%	2	3.1%	
Female	71	45.2%	84	53.5%	2	1.3%	

Race							0.677
African American	19	40.4%	27	57.4%	1	2.1%	
Native American/Aleutian/Eskimo	0	0.0%	1	100.0%	0	0.0%	
Hispanic	47	32.4%	95	65.5%	3	2.1%	
Asian	0	0.0%	3	100.0%	0	0.0%	
White	3	50.0%	3	50.0%	0	0.0%	
Other	4	21.1%	15	78.9%	0	0.0%	

Religion							0.708
Catholic	44	33.3%	86	65.2%	2	1.5%	
Jewish	1	100.0%	0	0.0%	0	0.0%	
Buddhist	0	0.0%	2	100.0%	0	0.0%	
Protestant	0	0.0%	3	100.0%	0	0.0%	
No Religion	11	37.9%	17	58.6%	1	3.4%	
Muslim	2	25.0%	6	75.0%	0	0.0%	
Jehovah's Witness	0	0.0%	3	100.0%	0	0.0%	
Other	15	38.5%	23	59.0%	1	2.6%	
Blank	0	0.0%	4	100.0%	0	0.0%	

Mother's completed level of education							0.031
Less than high school	25	41.0%	36	59.0%	0	0.0%	
High school/GED	19	22.9%	62	74.7%	2	2.4%	
College	21	47.7%	23	52.3%	0	0.0%	
Professional degree	4	22.2%	13	72.2%	1	5.6%	
Other	1	16.7%	5	83.3%	0	0.0%	
Blank	3	33.3%	5	55.6%	1	11.1%	

Father's completed level of education							0.287
<high school	25	37.9%	41	62.1%	0	0.0%	
High school/GED	24	31.2%	50	64.9%	3	3.9%	
College	7	26.9%	18	69.2%	1	3.8%	
Professional degree	1	10.0%	9	90.0%	0	0.0%	
Other	6	54.5%	5	45.5%	0	0.0%	
Blank	10	32.3%	21	67.7%	0	0.0%	

Has an older sibling who received the HPV vaccine							1
Yes	44	34.1%	83	64.3%	2	1.6%	
No	27	34.6%	50	64.1%	1	1.3%	
Blank	0	0.0%	1	1.0%	0	0.0%	
Unclear	2	15.4%	10	76.9%	1	7.7%	

Has a younger sibling who received the HPV vaccine							1
Yes	40	33.1%	79	65.3%	2	1.7%	
No	29	34.1%	55	64.7%	1	1.2%	
Blank	0	0.0%	1	100.0%	0	0.0%	
Unclear	4	28.6%	9	64.3%	1	7.1%	

Heard of cervical cancer							<0.001
Yes	69	41.1%	97	57.7%	2	1.2%	
No	4	7.5%	47	88.7%	2	3.8%	
Heard of Pap screening							0.003
Yes	44	42.7%	56	54.4%	3	2.9%	
No	28	23.9%	88	75.2%	1	0.1%	

Understand the goal of Pap screening							0.003
Yes	29	50.9%	27	47.4%	1	1.8%	
No	43	26.7%	115	71.4%	3	1.9%	

Heard of sexually transmitted disease							0.241
Yes	69	34.2%	130	64.4%	3	1.5%	
No	4	21.1%	14	73.7%	1	5.3%	

Have had other vaccines							<0.001
Yes	35	72.9%	12	25.0%	1	2.1%	
No	33	22.6%	112	76.7%	1	0.7%	
Do not know	2	16.7%	8	66.7%	2	16.7%	

Believe at risk for abnormal Pap/cervical cancer							0.333
Yes	8	57.1%	6	42.9%	0	0.0%	
No	43	31.2%	92	66.7%	3	2.2%	
Do not know	16	28.6%	39	69.6%	1	1.8%	

**Table 3:** Willingness to receive the HPV vaccine among adolescent who have not yet been vaccinated.

	Yes		No		Do not know		*P*
	#	%	#	%	#	%	
Language							0.873
English	71	52.2%	17	12.5%	48	35.3%	
Spanish	3	42.9%	1	14.3%	3	42.9%	

Age (mean)	74	1605.00%	18	16.06			0.997

Gender							0.126
Male	25	42.4%	10	16.9%	24	40.7%	
Female	49	58.3%	8	9.5%	27	32.1%	

Race							0.062
African American	15	55.6%	0	0.0%	12	44.4%	
Native American/Aleutian/Eskimo	0	0.0%	1	100.0%	0	0.0%	
Hispanic	50	52.6%	13	13.7%	32	33.7%	
Asian	0	0.0%	0	0.0%	2	100.0%	
White	1	33.3%	1	33.3%	1	33.3%	
Other	8	53.3%	3	20.0%	4	26.7%	

Religion							0.569
Catholic	48	55.8%	10	11.6%	28	32.6%	
Buddhist	0	0.0%	0	0.0%	1	100.0%	
Protestant	1	33.3%	1	33.3%	1	33.3%	
No Religion	10	58.8%	2	11.8%	5	29.4%	
Muslim	1	16.7%	1	16.7%	4	66.7%	
Jehovah's Witness	1	33.3%	1	33.3%	1	33.3%	
Other	11	47.8%	3	13.0%	9	39.1%	
Blank	2	50.0%	0	0.0%	2	50.0%	

Mother's completed level of education							0.094
<high school	21	60.0%	3	8.6%	11	31.4%	
High school/GED	35	56.5%	9	14.5%	18	29.0%	
College	10	43.5%	2	8.7%	11	47.8%	
Professional degree	7	53.8%	3	23.1%	3	23.1%	
Other	0	0.0%	0	0.0%	5	100.0%	
Blank	1	20.0%	1	20.0%	3	60.0%	

Father's completed level of education							0.204
<high school	25	62.5%	2	5.0%	13	32.5%	
High school/GED	21	42.0%	8	16.0%	21	42.0%	
College	6	33.3%	3	16.7%	9	50.0%	
Professional degree	4	44.4%	3	33.3%	2	22.2%	
Other	3	60.0%	0	0.0%	2	40.0%	
Blank	15	71.4%	2	9.5%	4	19.0%	

Has an older sibling who received the HPV vaccine							0.683
Yes	41	49.4%	13	15.7%	29	34.9%	
No	26	52.0%	5	10.0%	19	38.0%	
Blank	1	100.0%	0	0.0%	0	0.0%	
Unclear	6	66.7%	0	0.0%	3	33.3%	

Has a younger sibling who received the HPV vaccine							0.183
Yes	41	51.9%	7	8.9%	31	39.2%	
No	26	47.3%	11	20.0%	18	32.7%	
Blank	1	100.0%	0	0.0%	0	0.0%	
Unclear	6	75.0%	0	0.0%	2	25.0%	

Heard of cervical cancer							0.111
Yes	55	56.7%	13	13.4%	29	29.9%	
No	19	41.3%	5	10.9%	22	47.8%	
Heard of Pap screening							0.131
Yes	33	58.9%	6	10.7%	17	30.4%	
No	41	47.1%	12	13.8%	34	39.1%	

Know the use of Pap							0.451
Yes	16	59.3%	4	14.8%	7	25.9%	
No	56	49.1%	14	12.3%	44	38.6%	

Heard of sexually transmitted disease							0.447
Yes	69	53.5%	16	12.4%	44	34.1%	
No	5	35.7%	2	14.3%	7	50.0%	

Have had other vaccines							0.319
Yes	9	75.0%	1	8.3%	2	16.7%	
No	53	47.7%	17	15.3%	41	36.9%	
Do not know	6	50.0%	0	0.0%	6	50.0%	

Believe at risk for abnormal Pap/cervical cancer							0.3
Yes	3	50.0%	2	33.3%	1	16.7%	
No	46	50.0%	13	14.1%	33	35.9%	
Do not know	21	55.3%	2	5.3%	15	39.5%	
